# Melanoma in Adolescents and Young Adults: Evaluation of the Characteristics, Treatment Strategies, and Prognostic Factors in a Monocentric Retrospective Study

**DOI:** 10.3389/fonc.2021.725523

**Published:** 2021-09-16

**Authors:** Paolo Del Fiore, Irene Russo, Beatrice Ferrazzi, Alessandro Dal Monico, Francesco Cavallin, Angela Filoni, Saveria Tropea, Francesco Russano, Claudia Di Prata, Alessandra Buja, Alessandra Collodetto, Romina Spina, Sabrina Carraro, Rocco Cappellesso, Lorenzo Nicolè, Vanna Chiarion-Sileni, Jacopo Pigozzo, Luigi Dall’Olmo, Marco Rastrelli, Antonella Vecchiato, Clara Benna, Chiara Menin, Daniela Di Carlo, Gianni Bisogno, Angelo Paolo Dei Tos, Mauro Alaibac, Simone Mocellin

**Affiliations:** ^1^ Soft-Tissue, Peritoneum and Melanoma Surgical Oncology Unit, Veneto Institute of Oncology - IOV IRCCS, Padua, Italy; ^2^ Division of Dermatology, Department of Medicine (DIMED), University of Padua, Padua, Italy; ^3^ Postgraduate School of Occupational Medicine, University of Verona, Verona, Italy; ^4^ Department of Medicine, University of Padua School of Medicine and Surgery, Padua, Italy; ^5^ Independent Statistician, Solagna, Italy; ^6^ Department of Cardiological, Thoracic, Vascular Sciences and Public Health, University of Padua, Padua, Italy; ^7^ Pathological Anatomy Unit, University Hospital of Padua, Padua, Italy; ^8^ Department of Medicine (DIMED), Unit of Pathology & Cytopathology, University of Padua, Padua, Italy; ^9^ Unit of Surgical Pathology & Cytopathology, Ospedale dell’Angelo, Mestre, Italy; ^10^ Melanoma Oncology Unit, Veneto Institute of Oncology IOV-IRCCS, Padua, Italy; ^11^ Department of Surgery, Oncology and Gastroenterology (DISCOG), University of Padua, Padua, Italy; ^12^ Immunology and Diagnostic Molecular Oncology Unit, Veneto Institute of Oncology IOV-IRCCS, Padua, Italy; ^13^ Hematology/Oncology Division, Department of Women’s and Children’s Health, University of Padua, Padua, Italy

**Keywords:** melanoma, skin cancer, AYA, adolescent and young adult oncology, adolescent and young adult melanoma, survival, incidence, melanoma surgical treatment

## Abstract

The “Veneto Cancer Registry” records melanoma as the most common cancer diagnosed in males and the third common cancer in females under 50 years of age in the Veneto Region (Italy). While melanoma is rare in children, it has greater incidence in adolescents and young adults (AYA), but literature offers only few studies specifically focused on AYA melanoma. The aim of this study was to describe the characteristics, surgical treatment, and prognosis of a cohort of AYA melanoma in order to contribute to the investigation of this malignancy and provide better patient care. This retrospective cohort study included 2,752 Caucasian patients (702 AYA and 2,050 non-AYA patients) from the Veneto Region who were over 15 years of age at diagnosis, and who received diagnosis and/or treatment from our institutions between 1998 and 2014. Patients were divided in adolescents and youth (15-25 years), young adults (26-39 years) and adults (more than 39 years) for the analysis. We found statistically significant differences in gender, primary site, Breslow thickness, ulceration, pathologic TNM classification (pTNM) stage and tumor subtype among the age groups. Disease-specific survival and disease-free survival were also different among the age groups. Our findings suggest that the biological behavior of melanoma in young people is different to that in adults, but not such as to represent a distinct pathological entity. Additional and larger prospective studies should be performed to better evaluate potential biological and cancer-specific differences between AYAs and the adult melanoma population.

## 1 Introduction

The incidence of melanoma is continuously increasing in both adult and pediatric population around the world (1, 2). Although melanoma is rare in pediatric patients, the risk of developing melanoma grows significantly in adolescents and young adults, and represents the second most common type of cancer in this age group (3–5). The literature on melanoma offers very few studies specifically addressing adolescents and young adults (AYA). Of note, previous studies presented clinical and prognostic differences between melanoma diagnosed in adolescents, young adults and adults (6, 7). Specific clinical practice guidelines for the treatment of melanoma in AYA do not exist, and current management is similar to melanoma in adults.

This study compared characteristics, surgical treatment, and prognosis in a cohort of melanoma patients according to the age at diagnosis, with the purpose of underling potential differences in terms of tumor characteristics and prognosis between melanoma in AYA and melanoma in adults (non-AYA).

## 2 Materials and Methods

### 2.1 Study Design

This is a retrospective cohort study of patients who were diagnosed and/or treated for Melanoma of the skin between 1998-2014 at the Veneto Institute of Oncology (IOV) and at the University Hospital of Padua (UHP).

### 2.2 Material

The study included all 2,752 patients aged ≥15 years and living in the Veneto Region, who were diagnosed and/or treated for Primary Melanoma of the skin between 1998-2014 at the Veneto Institute of Oncology (IOV) and at the University Hospital of Padua (UHP). IOV and UHP are level III referral centers which are located in Northeastern Italy. Most patients are referred for diagnosis and/or first-line treatment, while some patients are referred for disease progression after being treated at local level II centers.

### 2.3 Diagnosis and Treatment

Melanoma was diagnosed according to the histopathology and immunohistochemistry of the lesion biopsy. Tumor stage was defined according to the eighth version of the American Joint Committee on Cancer (AJCC) staging system (8), effective from January 2018. All diagnoses before January 2018 were re-staged according to the last version of the staging system.

The surgical treatment included wide excision (WE) of the primary lesion, sentinel lymph node biopsy (SNB) and/or regional lymph node dissection. Patients with locoregional primary melanoma underwent WE, followed by complete lymph node dissection (LND) in clinical node-positive patients. Sentinel node biopsy was performed concurrently with WE in patients with primary lesions.

Follow-up visits were performed every three to four months for the first three years, every six months for up to five years, and every year thereafter.

Disease progression included regional recurrences, in-transit metastases, lymph node metastases, and distant metastases.

### 2.4 Data Collection

All data were extracted from a local database. Demographics included age at diagnosis, sex and family history, while tumor information included subtype of melanoma (such as acral lentiginous melanoma, lentigo maligna melanoma, nodular melanoma, superficial spreading melanoma, spitzoid melanoma, nevoid melanoma, pagetoid melanoma, polypoid melanoma, desmoplastic melanoma, minimal deviation melanoma and neurotropic melanoma) primary site, Breslow thickness, ulceration, mitotic rate, and pTNM stage.

Follow-up information was extracted from scheduled visits. Follow‐up was calculated from the date of diagnosis to December 31, 2019. Disease-specific survival was calculated from date of diagnosis to date of disease-related death, or date of last visit/disease-unrelated death. Disease-free survival was calculated in patients with primary melanoma from date of diagnosis to date of recurrence, or date of last visit/death. Recurrence was defined as regional recurrences, in-transit metastases, lymph node metastases or distant metastases.

### 2.5 Statistical Analysis

Continuous data were summarized as median and interquartile range (IQR). The patient cohort was divided into three age groups: adolescents and youth (15-25 years), young adults (26-39 years) and adults (more than 39 years). Categorical data were compared between age groups using the Fisher’s exact test, while the Kruskal-Wallis test was used for continuous data. Survival estimates were calculated using the Kaplan-Meier method and compared among age groups using log-rank test (unadjusted analysis) and Cox regression models with pTNM stage as additional independent variable (adjusted analysis). Effect sizes were reported as hazard ratio (HR) with 95% confidence interval (CI). The limited sample size and availability of immunohistochemistry data did not allow any meaningful multivariable analyses. All tests were two-sided and a p-value of less than 0.05 was considered statistically significant. Statistical analyses were performed using R software version 4.1 (R Foundation for Statistical Computing, Vienna, Austria) (9).

### 2.6 Ethics Considerations

The study was approved by the local Ethics Committee (number 2/2020). The study was conducted according to Helsinki Declaration principles, and all patients gave their consent to have their anonymized data used for scientific purpose.

## 3 Results

### 3.1 Patients

This analysis included 2,752 Caucasian patients aged ≥15 years, involving 76 (2.8%) adolescents and youth (median 22 years, IQR 20-24), 626 (22.7%) young adults (median 34 years, IQR 31-37) and 2,050 (74.5%) adults (median 54 years, IQR 48-68). Patient characteristics according to age classes are outlined in **Table 1**. Sex, primary tumor site, Breslow thickness, ulceration, number of mitoses, tumor stage and sub-type differed among the age classes.

**Table 1:** Patient and tumor characteristics in 2,752 patients aged ≥15 years and living in the Veneto Region who were diagnosed and/or treated for Melanoma of the skin between 1998-2014 at the Veneto Institute of Oncology and at the University Hospital of Padua (Italy): comparison between age classes (15-25 years, 26-39 years, 40 years or older).

	Adolescents and youth (15-25 years)	Young adults (26-39 years)	Adults (40 years or older)	p-value
N	76	626	2050	–
Sex:				**<0.0001**
Female	43 (56.6)	389 (62.1)	1010 (49.3)	
Male	33 (43.4)	237 (37.9)	1040 (50.7)	
Primary site:				**0.0003**
Acral	2 (2.6)	16 (2.6)	137 (6.7)	
Head/neck	2 (2.6)	32 (5.1)	150 (7.3)	
Upper limb	7 (9.2)	89 (14.2)	295 (14.4)	
Trunk	47 (61.8)	318 (50.8)	947 (46.2)	
Lower limb	18 (23.6)	171 (27.3)	521 (25.4)	
Breslow ^ab^	0.63 (0.39-1.20)	0.57 (0.35-1.09)	0.75 (0.39-1.90)	**<0.0001**
Ulceration:				**0.04**
Absent	56 (73.7)	508 (81.1)	1561 (76.1)	
Present	16 (21.0)	100 (16.0)	435 (21.2)	
Unknown	4 (5.3)	18 (2.9)	54 (2.6)	
Mitotic rate(per mm^2^) ^ac^	1 (0-2)	1 (0-2)	2 (0-4)	**0.02**
pTNM stage:				**0.0005**
I	55 (72.4)	470 (75.1)	1387 (67.7)	
II	10 (13.1)	64 (10.2)	337 (16.4)	
III	11 (14.5)	90 (14.4)	326 (15.9)	
IV	0 (0.0)	2 (0.3)	0 (0.0)	
pT:				**<0.0001**
1a-b, 2a-b	64 (84.2)	534 (85.3)	1562 (76.2)	
3a-b, 4a-b	12 (15.8)	92 (14.7)	488 (23.8)	
pN:				0.63
0	65 (85.5)	536 (85.6)	1724 (84.1)	
1-3	11 (14.5)	90 (14.4)	326 (15.9)	
pM:		624 (99.7)		0.11
0	76 (100.0)	2 (0.3)	2050 (100.0)	
1a-d	0 (0.0)		0 (0.0)	
Subtype: ^d^				**<0.0001**
ALM^*^	1 (1.5)	7 (.2)	72 (3.5)	
LMM^*^	0 (0.0)	2 (0.3)	37 (1.8)	
NM^*^	9 (12.7)	74 (12.9)	335 (16.3)	
SSM^*^	53 (74.6)	469 (81.6)	1541 (75.2)	
Other**	8 (11.2)	23 (4.0)	65 (3.2)	

Data expressed as n (%) or ^a^ median (IQR). Data not available in ^b^70, ^c^15, ^d^56 patients. ^*^ALM, Acral lentiginous melanoma; LMM, lentigo maligna melanoma; NM, nodular melanoma; SSM, superficial spreading melanoma. **Spitzoid Melanoma, Nevoid melanoma, pagetoid melanoma, polypoid melanoma, desmoplastic melanoma, minimal deviation melanoma and neurotropic melanoma.

The bold values are statistically significant.

### 3.2 Treatment of Primary Melanoma

All patients underwent WE, while SNB was performed in 1,412 patients and LND in 394. Treatment according to age classes is shown in **Table 2**. The number of excised sentinel lymph nodes (SLNs) differed among age classes, while the number of positive sentinel lymph nodes and the number of positive dissected lymph nodes were the same.

**Table 2:** Treatment of primary Melanoma of the skin in 2,752 patients aged ≥15 years and living in the Veneto Region who were diagnosed and/or treated for Melanoma between 1998-2014 at the Veneto Institute of Oncology and at the University Hospital of Padua (Italy): comparison between age classes (15-25 years, 26-39 years, 40 years or older).

	Adolescents and youth (15-25 years)	Young adults (26-39 years)	Adults (40 years or older)	p-value
N	76	626	2050	–
Patients who underwent WE	76 (100.0)	626 (100.0)	2050 (100.0)	**-**
Patient with positive SNB or clinical detection	10/75 (13.3)	83/612 (13.6)	272/2050 (13.3)	0.98
Excised sentinel nodes ^a^	2 (2-4)	2 (1-3)	2 (1-3)	**0.002**
Positive sentinel nodes ^a^	0 (0-0)	0 (0-0)	0 (0-0)	0.33
Positive dissected lymph node ^a^	0 (0-6)	0 (0-1)	0 (1-3)	0.22

Data expressed as n (%) or ^a^ median (IQR). WE, Wide excision; SNB, sentinel lymph node biopsy. SNB was conducted in 41/76 adolescents, 324/626 young adults and 1047/2050 adults. Clinical detection: 0 adolescents, 9 young adults and 43 adults. Positive SNB: 10 adolescents, 74 young adults and 229 adults. Total lymphadenectomy: 10 adolescents, 83 young adults, 272 adults. The bold values are statistically significant.

#### 3.2.1 Disease-Specific Survival

**Figure 1:**
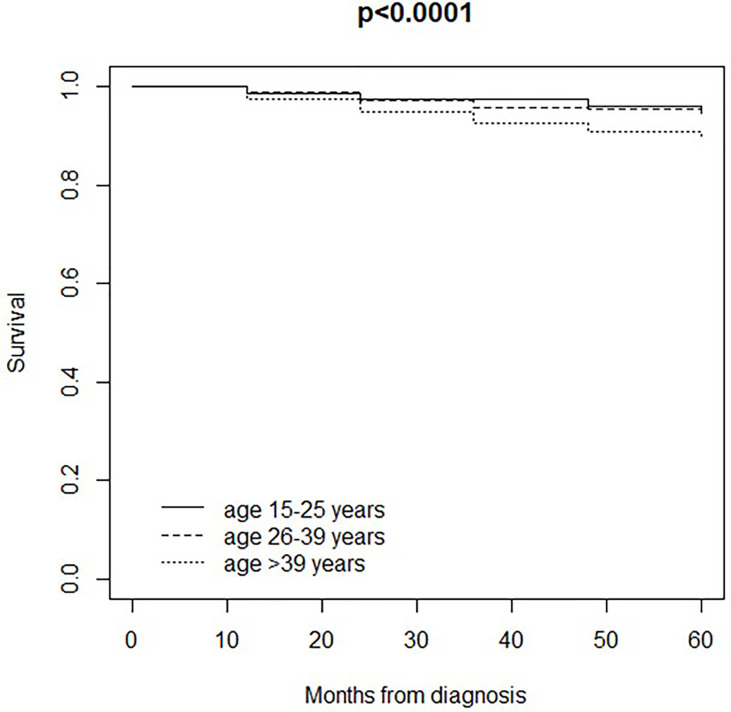
Disease-specific survival in 2,734 patients (18 patients were lost to follow-up) aged ≥15 years and living in the Veneto Region who were diagnosed and/or treated for Melanoma between 1998-2014 at the Veneto Institute of Oncology and at the University Hospital of Padua (Italy): comparison between age classes (15-25 years, 26-39 years, 40 years or older).

Median follow-up was 96 months (IQR 60-132). Overall, 312 patients died from the disease, and 211 patients died due to other causes (18 patients were lost to follow-up). 5-year disease-specific survival was 95% in patients aged 15-25 years, 95% in patients aged 26-39 years, and 90% in patients over 39 years (p<0.0001) (**Figure 1**). Adjusting for pTNM stage, patients aged 26-39 years had better disease-specific survival compared to patients over 39 years (HR 0.52, 95% CI 0.37 to 0.70; p<0.0001), while the difference between patients aged 15-25 years and patients over 39 years was not statistically significant (HR 0.46, 95% CI 0.19 to 1.12; p=0.09).

#### 3.2.2 Disease-Free Survival

393 patients experienced a clinical event during follow-up: local recurrence in 56 patients, regional lymph node metastasis in 128, regional skin/in-transit in 130, and distant metastasis in 167.

**Figure 2:**
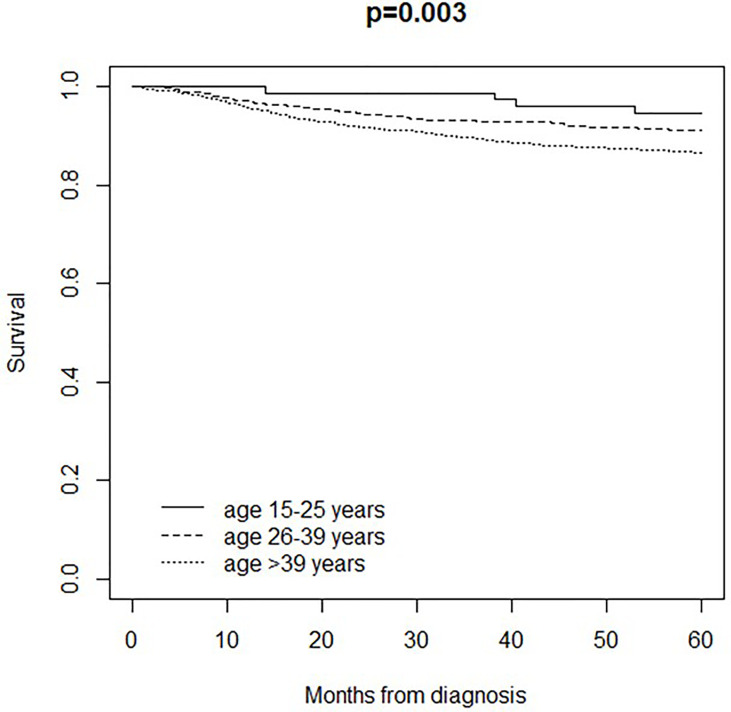
Disease-free survival in 2,734 patients (18 patients were lost to follow-up) aged ≥15 years and living in the Veneto Region who were diagnosed and/or treated for Melanoma between 1998-2014 at the Veneto Institute of Oncology and at the University Hospital of Padua (Italy): comparison between age classes (15-25 years, 26-39 years, 40 years or older).

5-year disease-free survival (i.e. survival until the occurrence of a clinical event or death/last visit) was 95% in patients aged 15-25 years, 91% in patients aged 26-39 years, and 87% in patients over 39 years (p=0.003) (**Figure 2**). Adjusting for pTNM stage, disease-free survival was better in patients aged 15-25 years (HR 0.42, 95% CI 0.19 to 0.95; p=0.04) and patients aged 26-39 years (HR 0.74, 95% CI 0.57 to 0.95; p=0.02) compared to patients over 39 years.

“Local recurrence”-free survival did not differ among age classes (p=0.20)

“Regional lymph node metastasis”- free survival differed among age classes (5-year survival: 98% in patients aged 15-25 years, 97% in patients aged 26-39 years, and 95% in patients aged over 39 years; p=0.02). Adjusting for pTNM stage, patients aged 26-39 years had better “regional lymph node metastasis”- free survival compared to patients over 39 years (HR 0.58, 95% CI 0.36 to 0.95; p=0.03), while the difference between patients aged 15-25 years and patients over 39 years was not statistically significant (HR 0.46, 95% CI 0.11 to 1.85; p=0.27).”Regional skin/in-transit” - free survival differed among age classes (5-year survival: 98% in patients aged 15-25 years, 98% in patients aged 26-39 years, 95% in patients aged over 39 years; p=0.003). Adjusting for pTNM stage, patients aged 26-39 years had better “regional skin/in-transit”- free survival compared to patients over 39 years (HR 0.44, 95% CI 0.26 to 0.75; p=0.002), while the difference between patients aged 15-25 years and patients over 39 years was not statistically significant (HR 0.42, 95% CI 0.10 to 1.72; p=0.23). “Distant metastasis” - free survival did not differ among age classes (p=0.06).

## 4 Discussion

There is currently a lack of data with regard to melanoma features and outcomes in AYA. A distinction should be made between AYA and older adult cancer in disease biology, treatment efficacy, and psychosocial barriers to care for patients. Moreover, patients aged 15-25 are a sub-category of AYA which is trapped in a medical gray area and may receive cancer treatment from pediatric or adult oncologists. Although this may not be perceived as an important aspect, treatment regimens for pediatric and adult cancer can lead to significant survival differences (10).

Overall, we found some differences in epidemiological, clinical, histopathological and prognostic features between AYA and non-AYA melanoma patients. Furthermore, some differences between adolescents and young adults also emerged.

We found more female patients in the AYA group than in the non-AYA. Females represented 56.6% of adolescents and youth (15-25 years old) and 62.1% of young adults (26-39 years old), while the proportion of males and females was similar among older adults. This finding is consistent with available literature (3), and could be explained by both biological and behavioral gender differences between young male and female patients (11). A strong endogenous estrogen exposure, due to early menarche associated with UV exposure during childhood, seems to play a crucial role in cutaneous melanoma development, and may explain the higher occurrence of melanoma in young females than in males (11).

AYA melanoma presented higher involvement of the trunk, while non-AYA melanoma were more common in the acral region, head/neck and upper limbs. On the other hand, the occurrence in lower limbs was similar among the age groups. Adolescent subjects, compared to young adults, had less involvement of the head/neck and upper limbs, while the trunk was the main affected site. These data mirror what is already known in the literature, namely, that the most affected sites are the trunk and lower limbs in young people and the head/neck and upper limbs increases among adults (3). However, the involvement of the trunk, upper limbs and head/neck in young adults was more similar to older adults than adolescents.

We found a different distribution of histological subtypes of melanoma between AYAs and older adults. Superficial spreading melanoma (SSM) was more common in young adults than in adults, but less common in adolescents than in adults. Nodular melanoma (NM), lentigo maligna melanoma (LMM) and acral lentiginous melanoma (ALM) were more common in adults. There was a considerable fraction of rare melanomas in adolescents these findings largely reflect what is known in the literature, namely, the greater presence of NM in older age, the presence of ALM almost exclusively in the elderly and rare melanomas in adolescent and young patients (12).

Moreover, we found that older adults were the subgroup with the worst pathological characteristics at the time of diagnosis. 21.2% of older adults presented ulceration, 32.3% had pTNM stage above the first, and the median Breslow thickness was 0.75 mm. At the time of diagnosis, melanoma in our cohort of patients was generally less advanced in AYAs than in adults. However, we found important differences between adolescents and young adults. Median Breslow thickness was 0.63 mm in adolescents and 0.57 mm in young adults; ulceration was found 21% in adolescents and 16% in young adults; a pTNM stage above the first was described in 27.6% of adolescents and in 24.9% of young adults. AYAs had lower Breslow thickness and lower pTNM stage at the time of diagnosis compared to adults, while there was no difference in regional lymph node involvement among age groups. Of note, adolescents had slightly worse stage characteristics than young adults.

It is important to emphasize that regional lymph node invasion (detected through positivity of the SNB or clinical positivity) was almost the same in the three age groups. Most pediatric melanoma studies suggested that the clinical history of melanoma in children and adolescents resembled that of adult disease (3, 4, 6, 7). As in adults, features such as ulceration, tumor thickness, and node involvement seemed to affect prognosis. Hence, in the absence of specific treatment guidelines, AYA melanoma is currently managed in the same way as non-AYA melanoma, though it is unclear whether it actually may have the same biological features as adult melanoma.

Data concerning the aggressiveness and prognosis of melanoma in AYAs are discordant in literature. Some studies reported that melanomas diagnosed in children and adolescents had higher Breslow thickness, greater tendency to regional lymph node invasion and, generally, a more advanced stage at the time of diagnosis compared to adults (13–17). On the other hand, it was also reported that young patients tended to have a better overall disease-specific survival than older adults (4, 15, 18).

In our study, disease-specific survival and disease-free survival were worse in older adults than in AYAs. Of note, most survival differences between younger and older age classes persisted after adjusting for tumor stage. This can be attributed to a diminished immune response with increased age, changes in host immune biology, and undertreatment due to medical comorbidities that may limit therapy with antineoplastic and biologic agents. The immune surveillance mechanism is one of the main factors which are hypothesized to account for better melanoma survival in the adolescents (19). Nonetheless, promoting skin cancer screening and public education (such as skin protection and self-examination awareness) is of utmost importance in patients of any age.

Regional lymph node metastasis-free survival and regional skin/in transit-free survival were different among age classes, with improved survival in young adults over adults, while the difference between adolescents and adults did not achieved statistical significance (likely due to the small number of adolescents in the study). Regional lymph node metastasis-free survival and regional skin/in transit-free survival have a substantial difference in survival regardless of patient’s age and involve a different therapeutic strategy.

The findings of this study should be interpreted considering his strengths and limitations. The strengths involve the completeness of information regarding epidemiological, clinical, histopathological and prognostic features, and the follow-up duration (at least 5 years for all patients). The limitations include the retrospective nature of the study and the absence of data regarding the analysis of mutational profiles and medical treatments for advanced melanoma. Furthermore, the small number of adolescents (which reflects melanoma epidemiology) may limit the generalizability of the findings for this subgroup.

Nonetheless, our study highlighted a sub-category of AYA aged 15-25 which may receive cancer treatment from pediatric or adult oncologists, with potential difference in survival outcome (10). The proportion of stage II-III melanoma among such patients suggest the need for adequate communication about prevention and awareness. Future research may confirm our results and explore the most appropriate and effective ways of implementing educational interventions among AYA aged 15-25.

## 5 Conclusion

Our findings show that AYA melanoma does not represent a distinct pathological entity and however there are differences they do not require a different therapeutic strategy because AYA melanoma has a clinical outcome comparable or better than melanoma in adults.

## Data Availability Statement

The datasets presented in this study can be found in online repositories. The names of the repository/repositories and accession number(s) can be found below: https://doi.org/10.5281/zenodo.4736486, 10.5281/zenodo.4736486.

## Ethics Statement

The studies involving human participants were reviewed and approved by Il Comitato Etico per la Sperimentazione Clinica (CESC) IOV. Written informed consent to participate in this study was provided by the participants’ legal guardian/next of kin.

## Author Contributions

Study concepts: PF, BF, AF, MA, and AV. Study design: PF, BF, CP, RS, and AC. Data acquisition: PF, VC, AB, AM, and JP. Quality control of data and algorithms: AM and FC. Data analysis and interpretation: PF, FC, AM, ST, FR, CB and MR. Statistical analysis: FC. Manuscript preparation: BF and PF. Manuscript editing: BF, PF, and IR. Manuscript review: FC, MA, SM, AB, VC-S, DC, GB, CM, and AD. All authors contributed to the article and approved the submitted version.

## Conflict of Interest

The authors declare that the research was conducted in the absence of any commercial or financial relationships that could be construed as a potential conflict of interest.

## Publisher’s Note

All claims expressed in this article are solely those of the authors and do not necessarily represent those of their affiliated organizations, or those of the publisher, the editors and the reviewers. Any product that may be evaluated in this article, or claim that may be made by its manufacturer, is not guaranteed or endorsed by the publisher.
