# Corrigendum: Melanoma in Adolescents and Young Adults (AYA): Evaluation of the Characteristics, Treatment Strategies, and Prognostic Factors in a Monocentric Retrospective Study

**DOI:** 10.3389/fonc.2021.793169

**Published:** 2021-10-27

**Authors:** Paolo Del Fiore, Irene Russo, Beatrice Ferrazzi, Alessandro Dal Monico, Francesco Cavallin, Angela Filoni, Saveria Tropea, Francesco Russano, Claudia Di Prata, Alessandra Buja, Alessandra Collodetto, Romina Spina, Sabrina Carraro, Rocco Cappellesso, Lorenzo Nicolè, Vanna Chiarion-Sileni, Jacopo Pigozzo, Luigi Dall’Olmo, Marco Rastrelli, Antonella Vecchiato, Clara Benna, Chiara Menin, Daniela Di Carlo, Gianni Bisogno, Angelo Paolo Dei Tos, Mauro Alaibac, Simone Mocellin

**Affiliations:** ^1^ Soft-Tissue, Peritoneum and Melanoma Surgical Oncology Unit, Veneto Institute of Oncology - IOV IRCCS, Padua, Italy; ^2^ Division of Dermatology, Department of Medicine (DIMED), University of Padua, Padua, Italy; ^3^ Postgraduate School of Occupational Medicine, University of Verona, Verona, Italy; ^4^ Department of Medicine, University of Padua School of Medicine and Surgery, Padua, Italy; ^5^ Independent Statistician, Solagna, Italy; ^6^ Department of Cardiological, Thoracic, Vascular Sciences and Public Health, University of Padua, Padua, Italy; ^7^ Pathological Anatomy Unit, University Hospital of Padua, Padua, Italy; ^8^ Department of Medicine (DIMED), Unit of Pathology & Cytopathology, University of Padua, Padua, Italy; ^9^ Unit of Surgical Pathology & Cytopathology, Ospedale dell’Angelo, Mestre, Italy; ^10^ Melanoma Oncology Unit, Veneto Institute of Oncology IOV-IRCCS, Padua, Italy; ^11^ Department of Surgery, Oncology and Gastroenterology (DISCOG), University of Padua, Padua, Italy; ^12^ Immunology and Diagnostic Molecular Oncology Unit, Veneto Institute of Oncology IOV-IRCCS, Padua, Italy; ^13^ Hematology/Oncology Division, Department of Women’s and Children’s Health, University of Padua, Padua, Italy

**Keywords:** melanoma, skin cancer, AYA, adolescent and young adult oncology, adolescent and young adult melanoma, survival, incidence, melanoma surgical treatment

In the published article, there was an error in **affiliation 1**. Instead of **^“^** ^1^Soft-Tissue, Peritoneum and Melanoma Surgical Oncology Unit, IOV- IRCCS, Padua, Italy”, it should be “^1^Soft-Tissue, Peritoneum and Melanoma Surgical Oncology Unit, Veneto Institute of Oncology - IOV IRCCS, Padua, Italy”.

In the published article, there was an error regarding the affiliation for **Antonella Vecchiato**. Instead of **affiliation 11**, she should have **affiliation 1**.

The authors apologize for these errors and state that this does not change the scientific conclusions of the article in any way. The original article has been updated.

## Publisher’s Note

All claims expressed in this article are solely those of the authors and do not necessarily represent those of their affiliated organizations, or those of the publisher, the editors and the reviewers. Any product that may be evaluated in this article, or claim that may be made by its manufacturer, is not guaranteed or endorsed by the publisher.

